# Effect of Textual Features on the Success of Medical Crowdfunding: Model Development and Econometric Analysis from the Tencent Charity Platform

**DOI:** 10.2196/22395

**Published:** 2021-06-11

**Authors:** Fuguo Zhang, Bingyu Xue, Yiran Li, Hui Li, Qihua Liu

**Affiliations:** 1 School of Information Management Jiangxi University of Finance and Economics Nanchang, Jiangxi China; 2 School of Management Zhejiang University of Technology Hangzhou, Zhejiang China; 3 School of Management Hainan University Haikou, Hainan China

**Keywords:** medical crowdfunding, textual features, project title, project details, fundraising success, theory of persuasion

## Abstract

**Background:**

Medical crowdfunding utilizes the internet to raise medical funds. Medical crowdfunding has developed rapidly worldwide; however, most medical crowdfunding projects fail to raise the targeted funds. Therefore, a very important research problem that has not received sufficient attention from the existing literature is identifying which factors affect the success of medical crowdfunding projects.

**Objective:**

The aim of this study was to examine the effect of textual features of medical crowdfunding projects on their success rate using 4903 real projects from the Tencent Charity platform, a well-known medical crowdfunding platform in China. In particular, according to Aristotle’s theory of persuasion, we divided the project text of medical crowdfunding into the project title and project details, which were analyzed from two perspectives (existence and extent) to explore their respective impacts.

**Methods:**

We established a research framework to meet our research goals. The process was divided into five main parts. We first collected data from Tencent Charity using Python programs and cleaned the datasets. Second, we selected variables and built the research model based on previous studies and the theory of persuasion. Next, the selected variables were extracted from the project text. We then performed econometric analysis using multiple regression analysis. Finally, we evaluated the results of econometric analysis to extract knowledge.

**Results:**

In the project title, the presence of the patient’s disease (*P*=.04) and occupation (*P*=.01) had a positive impact on the success rate of fundraising, whereas the presence of age (*P*<.001), gender (*P*=.001), and negative emotions (*P*=.04) had a negative impact. In the project details, the presence of the patient’s occupation (*P*=.01), monetary evidence (*P*=.02), and negative emotions (*P*=.04) played a positive role in the fundraising success rate, whereas the presence of age (*P*<.001) and positive emotions (*P*<.001) played a negative role. Moreover, in the project details, high-frequency monetary evidence (*P*=.02) and negative words (*P*=.02), as well as a short narrative length (*P*=.01) were conducive to succeeding in medical crowdfunding. Younger patients were more likely to obtain a higher success rate in medical crowdfunding. For patients whose occupations were national civil servant, professional skill worker, clerk, business and service worker, solider, child, student, and public-spirited person, the success rate of fundraising decreased sequentially.

**Conclusions:**

This study collected 4903 valid data from Tencent Charity, and identified which factors in the project text play an important role in the success rate of medical crowdfunding from the perspective of existence and extent. We found that in addition to the project details, the features of the project title also have an important impact on the success rate of fundraising. These findings provide important theoretical and managerial implications for medical crowdfunding.

## Introduction

### Background

With an aging population, the widespread prevalence of chronic diseases, a growing number of serious illnesses, and advances in medical technology, global health spending is rising rapidly. From 2018 to 2022, global health spending is expected to increase at an annual rate of 5.4%, representing a significant increase from 2.9% in 2013-2017 [1]. However, access to good health care such as through medical insurance and government subsidies is not available to all. Currently, half of the world’s population still lacks access to essential health services [2]. Additionally, even with partial health care, many medical conditions still incur high out-of-pocket medical costs, leading to significant financial stress on individuals and their families. In 2018, approximately 100 million people worldwide fell into extreme poverty because of out-of-pocket medical expenses [2].

In recent years, medical expenses in China have also increased rapidly. According to statistics from the National Health Commission, China’s medical expenses exceeded US $97.4 billion in 2019 [3]. Although China has established a relatively complete medical security system, many patients still have to pay large medical expenses themselves. China has established a multilevel medical security system with a basic medical insurance system as the main body, commercial insurance as a supplement, and social charity relief as a base. China has a basic medical insurance system, which receives subsidies for poor patients so they can be covered by insurance. As of January 2020, China’s basic medical insurance system covers more than 1.35 billion people, a coverage rate of approximately 97%, according to the National Healthcare Security Administration [4]. There are few people who currently purchase commercial insurance. As of October 2019, the number of people with major illness insurance policies in China did not exceed 100 million [5]. However, the current medical insurance system still has the following three problems. First, medical insurance reimbursement is restrictive, with reimbursements only provided for the hospital’s medical expenses, excluding nutrition and other expenses. Moreover, the expenses above this standard can be reimbursed only when the medical expenses reach the reimbursement standard. The reimbursement ratio ranges from 30% to 90%, and the maximum reimbursement limit is approximately US $40,000 [6]. Second, some drugs are not covered by reimbursement. For example, Neulasta/Peglasta is an imported drug that is effective in the treatment of cancer, but it is not on the reimbursement list. Finally, there are still more than 40 million Chinese people without medical insurance. Consequently, the existence of the above-mentioned problems has caused out-of-pocket medical expenses to remain high for many patients. There are still many families who suffer from poverty due to illness. As an example, Sohu News revealed that an 8-year-old girl in Tianjin, China was diagnosed with malignant neuroblastoma. Because of this illness, the family has spent more than US $60,000 and has accrued approximately US $30,000 of debt as a result. To cure this disease, follow-up treatment costs will reach nearly US $60,000 [7].

A unique and increasingly popular type of crowdfunding application, namely medical crowdfunding, is being used by many patients and their families to pay for medical bills. Medical crowdfunding has developed rapidly worldwide. GoFundMe, a US crowdfunding platform, raises US $650 million from approximately 250,000 medical crowdfunding campaigns each year [8]. A medical crowdfunding platform in China, Shuidichou, successfully provided fundraising services for more than 800,000 patients in economic distress as of the end of September 2018, raised more than US $1.4 billion, and aided over 340 million patients [9]. Other medical crowdfunding platforms have also grown rapidly in China. It is expected that medical crowdfunding worldwide will experience an annual growth rate of 25% [10]. However, the success rate is low for medical crowdfunding projects, as most of these campaigns fail to raise the target funds [11,12]. Berliner and Kenworthy [13] found that only 10% of the 200 raisers of medical funds randomly selected on the GoFundMe platform achieved their fundraising goals. Howard [14] pointed out that only a small proportion of medical fundraising campaigns are fully funded. The statistical data of 143,917 medical crowdfunding projects on Qschou.com show that only 7% of the projects successfully raised the target amount [15]. Therefore, a very important research goal is to identify the factors that affect the success rate of medical crowdfunding projects.

With the rapid development of medical crowdfunding, many studies have begun to examine this topic. Most researchers in this field have focused on the development of medical crowdfunding; the relationship with personal bankruptcy; and the fraud, privacy, and ethics issues that may be caused by medical crowdfunding [10,16-19]. However, little research has focused on the factors that impact the success of a medical crowdfunding project.

Medical crowdfunding is a new form of online crowdfunding. The role of the text on a fundraising page is important because project creators try to convince potential supporters with a compelling story [20]. Numerous studies have analyzed the effect of textual features on the success of online crowdfunding; however, determining whether these features also affect the success rate of medical crowdfunding projects is an extremely important and unsolved problem. Additionally, Aristotle’s theory of persuasion has been widely used in the online crowdfunding literature [20-22]. For example, Majumdar and Bose [20] studied the impact of narrative text on the possibility of pizza donation according to this theory. However, there is no literature related to the influence of textual features on the success of medical crowdfunding based on this theory at present. Therefore, we proposed research question 1: According to previous research and Aristotle’s theory of persuasion, do textual features have an important impact on the success of medical crowdfunding?

**Figure 1:**
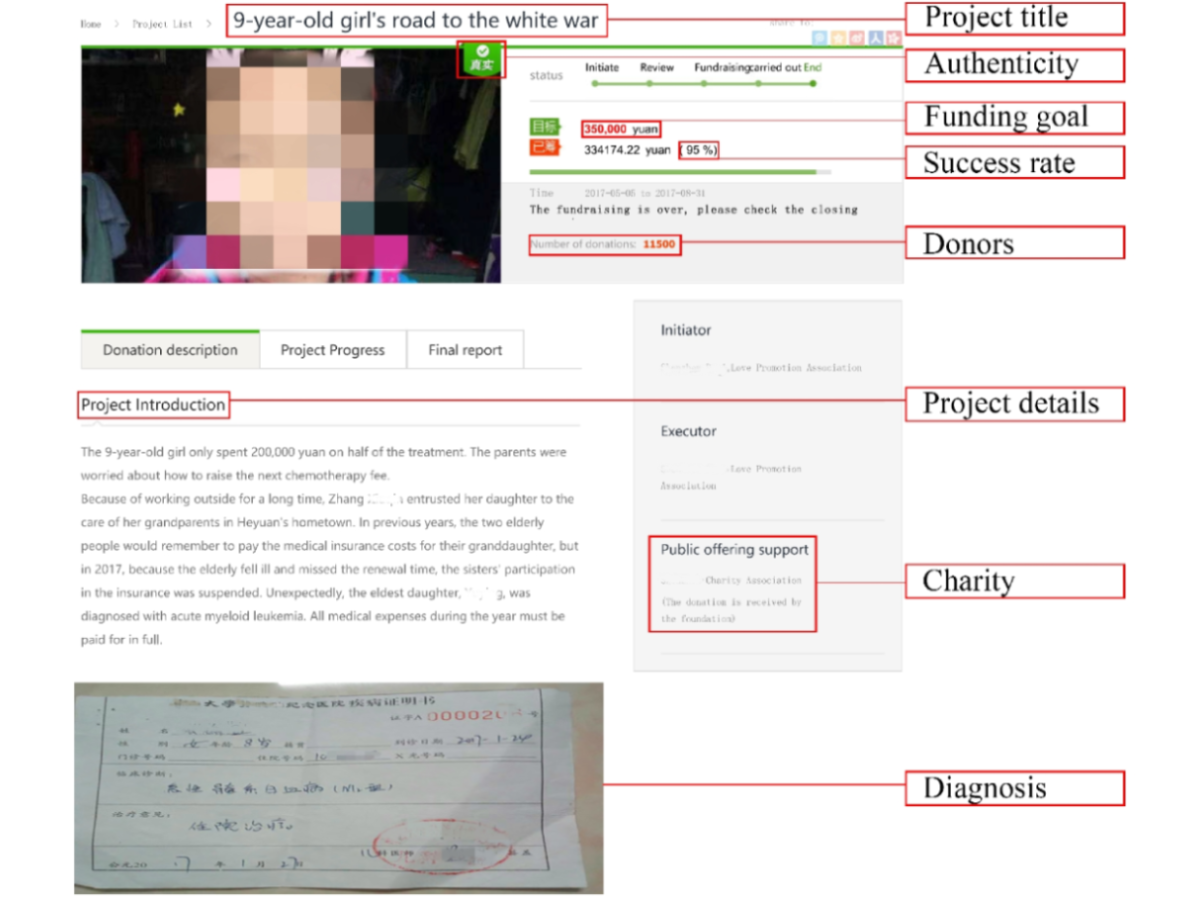
Screenshot of a medical crowdfunding project.

In medical crowdfunding, the project information generally consists of two parts: the project title and the project details, as shown in Figure 1. The project title is used to provide a brief overview of the crowdfunding project information using concise phrasing, and the project details are used to provide more complete information about the patient. Some studies have already investigated the impact of textual features on medical crowdfunding success; however, they focused on the impact of the text features of the project details and ignored the text features of the project title [23,24]. Medical crowdfunding projects are usually spread via social media in the form of links; therefore, potential donors often see the project title first. Accordingly, the textual features of the project title may affect the behavior of potential donors. If potential donors want to know more after reading the project title, they will further read the content of the project details. At this time, the text features of the project details may affect readers’ donation behavior. Therefore, it is possible that the text features of both the project title and project details will affect donors’ decision-making behavior, which in turn will affect the success of medical crowdfunding projects. Therefore, we proposed the following two research questions to explore the characteristics of the project title and project details that affect the success rate of medical crowdfunding projects:

- Research question 2: In the project title, what features can influence the success of medical crowdfunding?
- Research question 3: In the project details, what features can influence the success of medical crowdfunding?

### Literature Review

#### Medical Crowdfunding

Medical crowdfunding is a form of donation-based online crowdfunding. Online crowdfunding involves enterprises or individuals obtaining, through the internet, financial support from a large group of participants who provide small amounts of money [25]. There are many types of crowdfunding such as reward-based crowdfunding, equity-based crowdfunding, debt-based crowdfunding, and donation-based crowdfunding [26]. Reward-based crowdfunding involves individuals contributing comparatively small amounts of money to projects in return for some kind of reward. Rewards can range from a simple token such as a thank-you postcard to a production version of the crowdfunded product [27]. Equity-based crowdfunding is a mechanism that enables broad groups of investors to fund startup companies and small businesses in return for equity [28]. Debt-based crowdfunding is based on the relationship between a debtor and lender, and possesses a certain yield rate [29]. Donation-based crowdfunding, also called charitable crowdfunding, requires donors to devote themselves to the public good with no expectation of obtaining material rewards [30].

Medical crowdfunding aims to help those who are unable to pay their medical bills by enabling them to receive donations from others, and its text narrative usually involves many personal characteristics of the requester. In recent years, medical crowdfunding has attracted extensive attention within the academic community. Specifically, Snyder et al [18] analyzed 80 medical crowdfunding activities as examples of how Canadians prove to others that they should finance the medical expenses of requesters, and these reasons were evaluated according to relevant theories of ethics. Renwick and Mossialos [31] examined possible economic risks and benefits in the development of medical crowdfunding. Gonzales et al [16] demonstrated that medical crowdfunders often balance two aspects of a fundraising campaign: assistance from others and perceived privacy risks. Zenone and Snyder [19] summarized four possible types of fraud in medical crowdfunding: faking/exaggerating illnesses, impersonation, and misapplication of funds. Moore [17] studied an ethical issue involving the role of donors in medical crowdfunding. The research of Burtch and Chan [10] indicated that the number of individual bankruptcy filings in the United States is experiencing a trend of gradual decrease as a result of the rapid advancement of medical crowdfunding. Bassani et al [32] discussed the formation process and development direction of medical crowdfunding platforms, explored the relationship between medical crowdfunding and the national health system, and found that substitution effects function when the coverage rate of public health is low. After analyzing news reports on medical crowdfunding in the United States and Canada, Murdoch et al [33] discovered that there is little reportage of the negative descriptions of crowdfunding campaigns (4.76%), and found that the patients’ conditions were mostly cancer (49.11%) and rare diseases (36.01%).

In summary, previous studies have not directly elucidated the influencing factors of medical crowdfunding results but have rather probed the development of medical crowdfunding, and its impact on social and economic activities.

#### Influence of Textual Features on the Success of Online Crowdfunding

Crowdfunding projects are usually associated with a description of how the funds will be used [20]. In recent years, many studies have analyzed the impact of textual features on the success of online crowdfunding. Greenberg et al [34] found that some project information characteristics such as the project goal, project category, magnitude of the reward, project duration, whether the project is connected to social networks, and the number of words in the narrative can predict the success or failure of a reward-based crowdfunding campaign. Lukkarinen et al [28] discovered that the investment decisions of participants in equity-based crowdfunding are associated with the comprehensibility of the campaign and other easily grasped informational features to a great extent, with the traditional investment decision criteria used by venture capitalists or business angels having less importance. Lin [35] suggested that the language characteristics of the campaign requirements description, including readability, positivity, and deception cues, can predict the performance of the loan project.

Unlike other types of online crowdfunding, donation-based crowdfunding does not require financial returns, and the narrative usually contains little information about the economic benefits. Some scholars have explored the influence of textual features on the success of donation-based crowdfunding. Kshetri [36] pointed out that the identity of the fundraiser displayed in a donation-based crowdfunding campaign has a significant influence on donors’ behavior. Xu [37] examined the impact of three manifestations (video, pictures, and text) on the success rate of donation-based crowdfunding. Majumdar and Bose [20] analyzed 5671 real data items and found that donors’ behavior is influenced by many variables, including money-related words, the extent of use of negative emotions, and evidence relating to women and the authenticity of the information they provided. Additionally, Zubrickas [38] and Cason and Zubrickas [39] discussed how the inclusion of a refunding mechanism in the information can affect donors’ decision-making behavior. Sasaki [40] explained the effect of the amount of other people’s donations on subsequent behavior, and the empirical results showed that subsequent donors are more likely to increase their donations when the amount of the previous five donations increases. Cheng et al [41] proposed an optimal threshold in the number of donations provided by donation-based crowdfunding campaigns that promotes more donation activities.

Medical crowdfunding is a new form of donation-based crowdfunding. However, it is unknown whether the text characteristics of these projects affect their success rate. Only a few researchers have aimed to study this problem to date. Durand et al [23] indicated that medical crowdfunding for transplant surgery was more successful when the campaigns had longer descriptions, higher goal amounts, more positive emotions, and third-person narratives. Majumdar and Bose [20] discovered that reasonable and credible requests during a campaign can improve the chance of receiving donations. Xu and Wang [24] considered that certain messaging aspects such as constructing an image of being vulnerable and worthy of help, utilizing a tragic narrative with elements of Chinese traditional culture, and contrasting patients’ experiences before and after the illness can arouse the sympathy of potential donors and prompt them to donate to patients. However, these studies analyzed the impact of only a small number of textual features on medical crowdfunding from a specific theoretical perspective. Whether the large number of textual features found in the online crowdfunding literature has an impact on the success rate of medical crowdfunding remains an unresolved research question. Moreover, these previous studies focused only on the influence of the textual features of the project details and ignored the role of the textual features of the project title. However, the project title also embodies a wealth of information that may play a prominent role in the success rate of medical crowdfunding projects.

#### Theory of Persuasion and Online Crowdfunding

As an important channel for people to understand the needs of a medical crowdfunding project, the main expectation of text narration is to persuade readers to donate to the requester. The type of persuasive strategy that can be used to improve the persuasiveness of the text is particularly significant. Aristotle supposed that rhetoric signifies “the faculty of observing in any given case the available means of persuasion” and proposed the theory of persuasion. This theory mainly includes three important methods of persuasion: Ethos, Logos, and Pathos [42]. Ethos means that speakers should show some authentic and reliable content to gain psychological recognition and the trust of the audience. Logos means that speakers should use logical statements to convince others. Pathos means that speakers should display their emotional factors and arouse the emotions of the audience [42,43].

Aristotle’s theory of persuasion has also been applied to the field of online crowdfunding. Tirdatov [21] examined the 13 most-funded crowdfunding texts from Kickstarter, and found that all of them contained the three types of rhetorical styles mentioned in the theory of persuasion (ie, Ethos, Pathos, and Logos). Majumdar and Bose [20] studied the content factors of rationality, emotion, and credibility in text narratives, and found that the presence of rational and credible content in charity messages is positively related to the probability of receiving pizza donations, but this was not the case for the emotional content. Wang and Wu [22] explored the impact of photographic narratives on online medical crowdfunding campaigns. They found that Pathos (emotional-related) and Ethos (credible-related) photos have positive persuasion effects on medical crowdfunding performance, whereas Logos (rational-related) photos have a negative persuasion effect. However, the existing literature has not yet used the theory of persuasion to study the influence of persuasive strategies of textual features on the results of medical crowdfunding.

Complementing this line of existing research, this study empirically examined the effect of the textual features of medical crowdfunding projects on their success rate, taking 4903 real projects collected from the Tencent Charity platform in China as the research sample. Based on the theory of persuasion, we divided the appeals in the text into three categories (rational, emotional, and credibility appeals), explored them from the two dimensions of project title and project details, and analyzed them from two perspectives of existence and extent.

## Methods

### Study Design

To meet the research objectives, a research framework was developed. The process was divided into five main sections: (1) collecting and cleaning the dataset, (2) selecting the variables and building the research model, (3) extracting the variables, (4) performing econometric analysis, and (5) evaluating the results of the econometric analysis to extract knowledge.

### Collecting and Cleaning the Dataset

There are two types of medical crowdfunding in China: public fundraisers and individual appeals. The “Charity Law” promulgated in 2016 stipulates that for public fundraisers, patients or their families must post fundraising information on the crowdfunding platform prescribed by the Civil Affairs Department of the State Council with the help of philanthropic organizations. These projects are publicly available on the crowdfunding platform where information is easily obtained. Individual appeals are published and disseminated exclusively on social media such as microblogs and cannot be publicly displayed on crowdfunding platforms. Compared to individual appeals, the information on crowdfunding platforms is more complete and has higher credibility, and the platforms have certain specifications for fundraisers so that the process is more normalized. Hence, the datasets in this study were obtained mainly from public fundraisers.

Our data sample was obtained from the Tencent Charity platform, which is one of the largest donation-based crowdfunding platforms in China and provides easy access to information. Tencent Charity was initiated by the Tencent Company in 2007. As of January 2019, its cumulative fundraising amount had exceeded US $700 million. We collected data using a crawler program, which we developed based on the Python language. The program first uses a Python third-party library (“requests”) to obtain webpage data containing crowdfunding projects. It then uses regular expressions to parse and extract the required information, and finally saves the data in an Excel table. The pseudocode of the crawler program is provided in Multimedia Appendix 1.

We collected all disease crowdfunding projects displayed on Tencent Charity in January 2019. An initial total of 5400 projects were obtained. We deleted projects that were raising funds for certain groups through manual filtering, because the focus of this study was personal medical crowdfunding. The main process was as follows. First, two graduate students marked projects for which the donation objects were not individuals by reading crowdfunding text from the dataset. Second, a third student compared the results of the previous two students. If there was substantial divergence, the three students discussed it until they reached agreement. In the end, a final dataset containing information on 4903 medical crowdfunding projects containing both nontextual features (funding goal, fundraising amount, and donors) and textual features (project title and project details) was obtained. In the dataset, the average values of funding goal, fundraising amount, and number of donors were 149,673, 34,569, and 1467, respectively. We used the fundraising amount divided by the funding goal to calculate the project’s fundraising success rate. Overall, the success rate of medical crowdfunding projects was found to be relatively low, with an average success rate of 0.31.

### Selecting Variables and Building the Research Model

We first selected variables according to the previous literature on online crowdfunding, which showed that certain textual features play an important role in the success rate of online crowdfunding projects, as shown in Table 1. The main textual features that have been found to have a significant impact include disease description, patient age, patient gender, regional economic development level, patient occupation, emotional and economic descriptions, vivid language, inclusive language, money-related language, title length, project information quality, description elaborateness, video, images, number of updates, prior experience, and duration. These features constituted the candidate feature set of the crowdfunding texts. We then determined the variables related to medical crowdfunding based on this feature set, including textual and nontextual features (Table 1).

**Table 1:** Textual features affecting the success rate of online crowdfunding projects.

Reference	Online crowdfunding type	Research variables
Xu and Wang [24]	Donation-based crowdfunding	Illness narrative and the patients’ identity such as disease severity and financial plight, tragic narrative strategies, etc
Aleksina et al [44]	Donation-based crowdfunding	Disease characteristics, medical research characteristics, organizational characteristics, comments, length, platform, etc
Kusumarani and Zo [45]	Donation-based crowdfunding	Perceived financial resources, perceived information resources, political interest, negative perception toward political situation, online community engagement, etc
Liang et al [46]	Reward-based crowdfunding	Funders’ trust, project information quality, fundraiser’s ability, project type and funding level, gender, age, education, income, prior experiences, website familiarity, etc
Petitjean [47]	Reward-based crowdfunding	Goal, geography, photo, video, website, updates, comments, Facebook friends, shares, etc
Durand et al [23]	Donation-based crowdfunding	Disease category, narrative perspective, gender, patient age group, location, goal amount, cumulative sentiment, sentiment range, description length
Majumdar et al [20]	Donation-based crowdfunding	Title length, reciprocity phrases, gratitude, popularity, comments received, account age, past participation, authentic, text length, presence of image, presence of monetary evidence, presence of negative emotions, presence of female references, extent of monetary evidence, extent of negative emotions, extent of female references
Kim et al [48]	Reward-based crowdfunding	Identity disclosure, prior experience, number of comments, number of updates, description elaborateness, campaign duration, funding goal, country, category
Allison et al [49]	Reward-based crowdfunding	Log funding, requested funding, adopted group, identity, positive narrative tone, etc
Bi et al [50]	Reward-based crowdfunding	Goal, duration, introduction word count, video count, “like” count, number of reviews
Lukkarinen et al [28]	Equity-based crowdfunding	Number of investors, amount raised, team rating, markets rating, concept rating, etc
Gorbatai et al [51]	Reward-based crowdfunding	Money-related language, vivid language, positive emotion, inclusive language, campaign duration, video, images, length of the campaign text, campaign goal, etc
This study	Donation-based crowdfunding	Age, gender, disease, location, occupation, money-related words, negative emotions, positive emotions, authenticity, diagnosis, charities, length of the project title and project details, funding goal, donors

**Figure 2:**
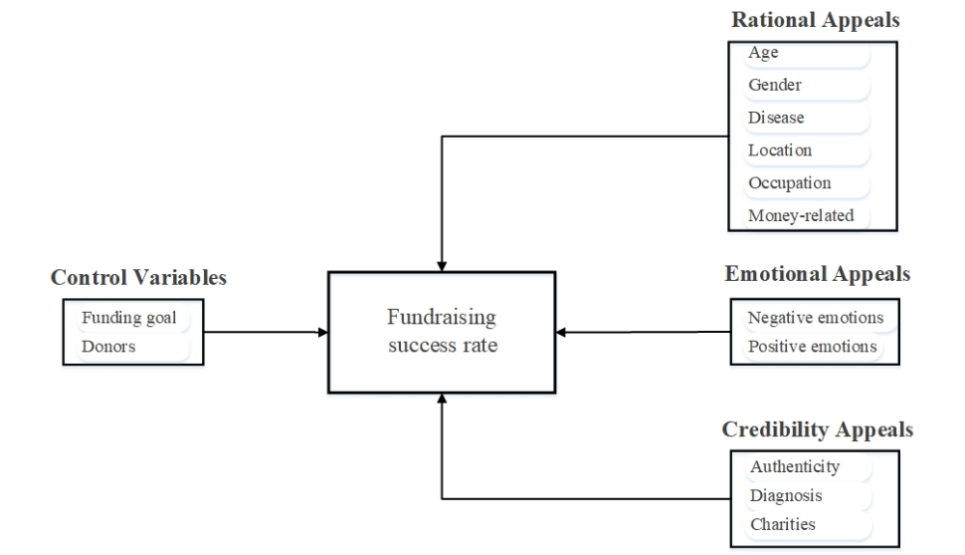
Research model.

We further built the research model based on Aristotle’s theory of persuasion, which includes three important methods of persuasion: Ethos, Logos, and Pathos [42]. Some previous studies have applied the theory of persuasion to the study of human behavior [20,22,43]. Majumdar and Bose [20] categorized content factors of online crowding into rational, emotional, and credibility based on this theory. Similarly, in this study, we divided the persuasion strategy of medical crowdfunding text into three categories: rational, emotional, and credibility appeals, corresponding to Logos, Pathos, and Ethos, respectively. Age, gender, disease, location, occupation, and money-related words all describe actual information about the patient, highlighting the characteristics of the donation objects, and therefore constitute rational appeals. Negative emotions and positive emotions express the feelings of the seeker and focus on appealing to the sympathy of potential donors, and therefore belong to emotional appeals. Authenticity, diagnosis, and charities are used to prove that the crowdfunding project is true and reliable, and are therefore classified as credibility appeals. Moreover, consistent with previous research [50], the nontextual features of funding goal and donors are regarded as control variables. The research model is schematically depicted in Figure 2.

### Extracting Variables

**Figure 3:**
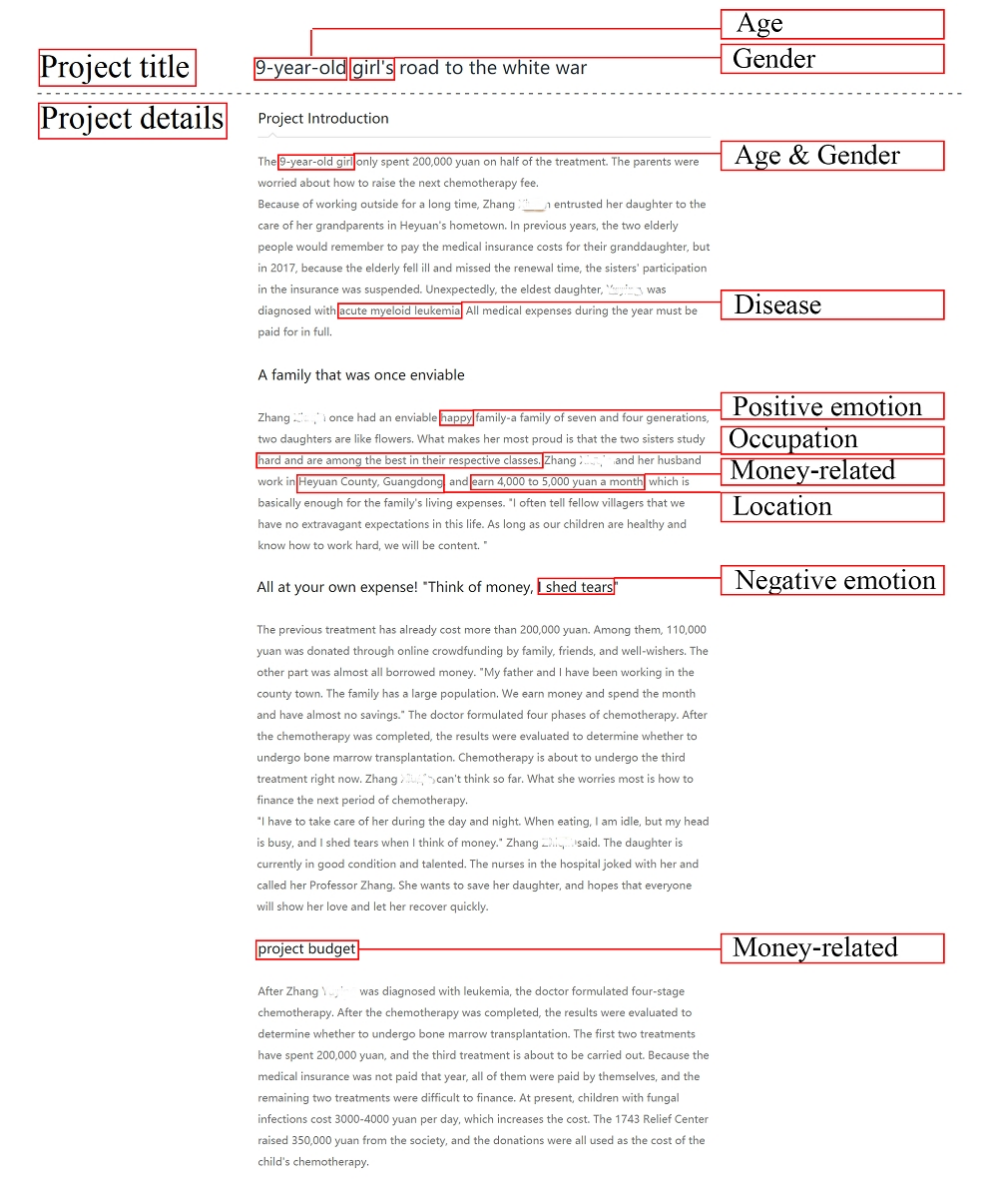
Textual features of a medical crowdfunding project.

In medical crowdfunding, some textual features appear in the project title, while others appear in the project details, and some may be reflected in both. Figure 3 shows a medical crowdfunding project from Tencent Charity with the textual features marked. We classified these variables into project title and project details. Moreover, a textual feature has two dimensions: whether the feature exists and the extent of the feature if it does exist. Majumdar and Bose [20] found that the presence of textual features (money-related terms and female references) and the extent of textual features (money-related terms, negative emotions, and female references) impact the success of donation-based crowdfunding. Therefore, we adopted a similar method to quantify all textual features in the dataset as existence variables or extent variables. Table 2 lists all of the variables considered with their descriptions.

**Table 2:** Description of the variables.

Variables	Description and measure
Success rate	Ratio of raised amount to target amount
Funding goal	The total amount to be raised by a fundraiser in a project (10,000)
Donors	Number of people contributing to a project
TPAge	Dummy variable; 1 if there is an age in the title, otherwise 0
TPGender	Dummy variable; 1 if there is a gender in the title, otherwise 0
TPDisease	Dummy variable; 1 if there is a disease description in the title, otherwise 0
TPOccupation	Dummy variable; 1 if there is an occupation in the title, otherwise 0
TPLocation	Dummy variable; 1 if there is a location in the title, otherwise 0
TPMoney	Dummy variable; 1 if there is a funding target in the title, otherwise 0
TPNWords	Dummy variable; 1 if there are negative words in the title, otherwise 0
TPPWords	Dummy variable; 1 if there are positive words in the title, otherwise 0
DPAge	Dummy variable; 1 if there is an age in the details, otherwise 0
DPGender	Dummy variable; 1 if there is a gender in the details, otherwise 0
DPDisease	Dummy variable; 1 if there is a disease description in the details, otherwise 0
DPOccupation	Dummy variable; 1 if there is an occupation in the details, otherwise 0
DPLocation	Dummy variable; 1 if there is a location in the details, otherwise 0
DPMoney	Dummy variable; 1 if there is a financial description in the details, otherwise 0
DPNWords	Dummy variable; 1 if there are negative words in the details, otherwise 0
DPPWords	Dummy variable; 1 if there are positive words in the details, otherwise 0
TLength	Number of words in the project title
TAge	Patient’s age in the project title
TGender	Patient’s gender in the project title
TDisease	Patient’s disease in the project title; 1 for mild disease and 0 for severe disease
TLocation	Patient’s location in the project title; sorted according to per capita GDP in 2018 of each city in descending order, and assign 1, 2, 3..., as shown in Multimedia Appendix 2
TOccupation	Patient’s occupation in the project title; classify patient’s occupation into nine categories and assign them values from 1 to 9, as shown in Multimedia Appendix 3
TNMWords	Number of mentions of money-related words in the project title
TNNWords	Number of negative emotion words in the project title
TNPWords	Number of positive emotion words in the project title
DLength	Number of words in the project details
DAge	Patient’s age in the project details
DGender	Patient’s gender in the project details
DDisease	Patient’s disease in the project details; 1 for mild disease and 0 for severe disease
DLocation	Patient’s location in the project details; sorted according to per capita GDP in 2018 of each city in descending order, and assign 1, 2, 3..., as shown in Multimedia Appendix 2
DOccupation	Patient’s occupation in the project details; classify patient’s occupation into nine categories and assign them values from 1 to 9, as shown in Multimedia Appendix 3
DNMWords	Number of mentions of money-related words in the project details
DNNWords	Number of negative emotion words in the project details
DNPWords	Number of positive emotion words in the project details
Authenticity	1 indicates that the project has been verified by the platform; otherwise, 0
Diagnosis	1 indicates that the project includes a hospital diagnosis certificate; otherwise, 0
Charities	1 indicates that charities are involved in the project; otherwise, 0

Automatic extraction and manual filtering were used to extract the textual features and corresponding variables. The patient’s age, gender, disease, occupation, and location, as well as money-related items were manually extracted because it was difficult to automatically process these variables, as the writing style of the text was personal and had no fixed or standardized format. For each feature, we determined the value of its existence variable and extent variable in the project title and project details. For example, for patient gender, we determined the values of four variables: *TPGender*, *DPGender*, *TGender*, and *DGender*. The manual extraction process took approximately 2 months to complete and was divided into two main stages. First, two graduate students independently extracted each feature by reading each individual text from the dataset. Second, a third student compared the results of the previous two students. If there was substantial divergence, the three students discussed it until they reached agreement. However, owing to the requirements of the Tencent Charity platform, all projects meet the credibility appeals. All of the crowdfunding projects we collected were certified by the platform, with hospital diagnostic reports and charities participation. Therefore, authenticity, diagnosis, and charities were set to a value of 1.

Other variables, including *TLength*, *DLength*, *TPNWords, TPPWords, TNNWords*, *TNPWords*, *DPNWords*, *DPPWords, DNNWords*, and *DNPWords*, were automatically extracted using Python and the HowNet lexicon, which is the Chinese version of WordNet and has been widely used for text mining and sentiment analysis [52-54]. First, we calculated the number of words in the project title and project details to obtain the values of the variables *TLength* and *DLength*. Next, we used a Chinese word segmentation component called Jieba in Python to segment the project title and project details, and then compared the words in the project title with the positive emotional vocabulary in the HowNet dictionary. If the match was successful, the value of the variable *TPPWords* was recorded as 1 and the value of the variable *TNPWords* was increased by 1. The values of other variables were calculated in the same manner.

### Building the Econometric Model

To better appreciate the effects of the textual features of the project title and project details, we used multiple regression analysis to construct five econometric models. Model 1 was the control-only model. Model 2 considered the presence variables of the project title and the control variables. Model 3 added the presence variables of the project details on the basis of Model 2. Model 4 considered the extent variables of the project title and the control variables. Model 5 added the extent variables of the project details on the basis of Model 4. Since the values of authenticity, diagnosis, and charities were all constant, indicating that there was no difference in the credibility appeals among the crowdfunding projects, these three variables were removed when building the econometric models.

- Model 1:
  Success rate = *α*_0_ + *α*_1_Funding goal + *α*_2_Donors + *ε*
- Model 2:
  Success rate = *α*_0_ + *α*_1_Funding goal + *α*_2_Donors + *α*_3_TPAge + *α*_4_TPGender + *α*_5_TPDisease + *α*_6_TPOccuption + *α*_7_TPLocation + *α*_8_TPMoney + *α*_9_TPWords + *α*_10_TPPWords + *ε*
- Model 3:
  Success rate = *α*_0_ + *α*_1_Funding goal + *α*_2_Donors + *α*_3_TPAge + *α*_4_TPGender + *α*_5_TPDisease + *α*_6_TPOccuption + *α*_7_TPLocation + *α*_8_TPMoney + *α*_9_TPWords + *α*_10_TPPWords + *α*_11_DPAge + *α*_12_DPGender + *α*_13_DPDisease + *α*_14_DPOccupation + *α*_15_DPLocation + *α*_16_DPMoney + *α*_17_DPNWords + *α*_18_DPPWords + *ε*
- Model 4:
  Success rate = *α*_0_ + *α*_1_Funding goal + *α*_2_Donors + *α*_3_TLength + *α*_4_TAge + *α*_5_TGender + *α*_6_TDisease + *α*_7_TOccupation + *α*_8_TLocation + *α*_9_TNMWords + *α*_10_TNNWords + *α*_11_TNPWords + *ε*
- Model 5:
  Success rate = *α*_0_ + *α*_1_Funding goal + *α*_2_Donors + *α*_3_TLength + *α*_4_TAge + *α*_5_TGender + *α*_6_TDisease + *α*_7_TOccupation + *α*_8_TLocation + *α*_9_TNMWords + *α*_10_TNNWords + *α*_11_TNPWords + *α*_12_DLength + *α*_13_DAge + *α*_14_DGender + *α*_15_DDisease + *α*_16_DOccupation + *α*_17_DLocation + *α*_18_DNMWords + *α*_19_DNNWords + *α*_20_DNPWords + *ε*

## Results

### Descriptive Statistics

Table 3 provides the descriptive statistics of the variables. *DPGender*, *DPDisease*, *Authenticity*, *Diagnosis*, and *Charities* were constant, and were therefore removed from subsequent analyses. The correlations between the variables are provided in Multimedia Appendix 4 and Multimedia Appendix 5. Given the overall lack of a strong correlation between the variables, we proceeded to the regression analysis.

**Table 3:** Descriptive statistics of variables.

Variables	Maximum	Minimum	Median	Mean	Variance
Success rate	1.47	0.00	0.12	0.31	0.14
Funding goal	350.00	1.00	10.00	14.97	241.11
Donors	77,760.00	5.00	311.00	1466.59	17,465,022.11
TPAge	1.00	0.00	0.00	0.14	0.12
TPGender	1.00	0.00	0.00	0.34	0.23
TPDescription	1.00	0.00	0.00	0.40	0.24
TPLocation	1.00	0.00	0.00	0.01	0.01
TPOccupation	1.00	0.00	0.00	0.10	0.09
TPMoney	1.00	0.00	0.00	0.005	0.005
TPNWords	1.00	0.00	0.00	0.03	0.03
TPPWords	1.00	0.00	0.00	0.19	0.15
DPAge	1.00	0.00	1.00	0.85	0.12
TPGender	1.00	1.00	1.00	1.00	0.00
TPDisease	1.00	1.00	1.00	1.00	0.00
DPLocation	1.00	0.00	1.00	0.84	0.13
DPOccupation	1.00	0.00	1.00	0.93	0.07
DPMoney	1.00	0.00	1.00	0.98	0.02
DPNWords	1.00	0.00	1.00	0.93	0.07
DPPWords	1.00	0.00	1.00	0.998	0.002
TLength	12.00	4.00	9.00	8.46	0.75
TAge	96.00	0.00	4.00	7.07	97.28
TGender	1.00	0.00	0.00	0.42	0.24
TDisease	1.00	0.00	0.00	0.02	0.02
TLocation	33.00	1.00	20.00	17.23	118.57
TOccupation	9.00	1.00	7.00	6.22	3.39
TNMWords	1.00	0.00	0.00	0.005	0.005
TNNWords	2.00	0.00	0.00	0.03	0.03
TNPWords	2.00	0.00	0.00	0.20	0.17
DLength	3785.00	121.00	1355.00	1370.20	218,283.22
DAge	96.00	0.00	8.00	13.15	196.38
DGender	1.00	0.00	1.00	0.60	0.24
DDisease	1.00	0.00	0.00	0.03	0.03
DLocation	43.00	1.00	18.00	18.29	74.85
DOccupation	9.00	1.00	7.00	6.73	2.11
DNMWords	22.00	0.00	3.00	3.87	6.58
DNNWords	30.00	0.00	4.00	4.35	12.11
DNPWords	68.00	0.00	16.00	16.96	59.48
Authenticity	1.00	1.00	1.00	1.00	0.00
Diagnosis	1.00	1.00	1.00	1.00	0.00
Charities	1.00	1.00	1.00	1.00	0.00

### Regression Results

We first estimated the effect of control variables on the success rate of project fundraising with Model 1. As shown in Table 4, the funding goal and number of donors were significantly related to the success rate. Specifically, the funding goal had a negative impact on the success rate of fundraising, and the coefficient of donors (19.00, *P*<.001) significantly and positively affected the success rate.

Based on these control variables, we then increased the number of existence variables in the project title with Model 2. As shown in Table 4, the effects of the funding goal and number of donors were significant in this model, coinciding with the findings for Model 1. Moreover, in rational appeals, the presence of a patient’s disease and occupation had a positive impact on the success rate of crowdfunding, whereas the presence of a patient’s age and gender had a negative impact. The presence of location and monetary evidence exhibited no significant effect. In emotional appeals, the coefficient of *TPNWords* (–3.83, *P*=.04) indicated that the presence of negative emotions in the project title played a negative role in the fundraising success rate. The presence of positive emotions was insignificant, which shows that the persuasive strategy of positive emotions had no effect.

Additionally, we added the factors of project details to Model 2 to build Model 3, so as to estimate the role of project details. As indicated in Table 4, the control variables still exhibited significant effects, consistent with Models 1 and 2. The significant variables in the project title were also the same as those found for Model 2. In rational appeals of the project details, the coefficients of *DPOccupation* and *DPMoney* were 3.07 (*P*=.01) and 5.01 (*P*=.02), respectively, reflecting that the presence of the patient’s occupation and money-related words had a positive relationship on the fundraising success rate. The presence of the patient’s age had a negative relationship and the presence of the patient’s location had no significant effect, which indicates that this information cannot persuade a donor. In emotional appeals, the presence of negative emotions was found to play a positive role in the success rate of fundraising, while the presence of positive emotions played a negative role, implying that the presence of negative emotions, rather than positive emotions, is more persuasive to donors.

**Table 4:** Regression results of existence variables.

Variables			Model 1 (adjusted R^2^=0.637)							Model 2 (adjusted R^2^=0.639)							Model 3 (adjusted R^2^=0.643)				
			*β*		*P* value		VIF^a^		*β*			*P* value		VIF		*β*			*P* value		VIF
**Control**																					
	Funding goal	–20.59		<.001		1.076		–20.51			<.001		1.114		–20.53			<.001		1.179	
	Donors	19.00		<.001		1.076		19.01			<.001		1.083		18.90			<.001		1.097	
**Title**																					
	TPAge	—^b^		—		—		–3.27			<.001		1.035		–2.96			<.001		1.051	
	TPGender	—		—		—		–2.29			.001		1.029		–2.29			.001		1.043	
	TPDisease	—		—		—		1.34			.04		1.032		1.41			.03		1.037	
	TPLocation	—		—		—		–4.90			.08		1.006		–5.45			.05		1.009	
	TPOccupation	—		—		—		2.67			.01		1.021		2.51			.02		1.025	
	TPMoney	—		—		—		–0.68			.88		1.006		–1.85			.68		1.011	
	TPNWords	—		—		—		–3.83			.04		1.008		–3.76			.04		1.008	
	TPPWords	—		—		—		–1.02			.21		1.015		–1.02			.21		1.017	
**Details**																					
	DPAge	—		—		—		—			—		—		–4.36			<.001		1.039	
	DPLocation	—		—		—		—			—		—		–1.20			.17		1.031	
	DPOccupation	—		—		—		—			—		—		3.07			.01		1.041	
	DPMoney	—		—		—		—			—		—		5.01			.02		1.004	
	DPNWords	—		—		—		—			—		—		2.55			.04		1.042	
	DPPWords	—		—		—		—			—		—		–34.65			<.001		1.013	

^a^VIF: variance inflation factor.

^b^—: not included in model.

Furthermore, we estimated the extent variables of the project title and project details to explore how the extent of a feature, if it exists, affects the fundraising results. Table 5 demonstrates the regression results for the extent variables of the project title and the control variables in Model 4. The funding goal exhibited a significant negative impact and the number of donors exhibited a significant positive effect, consistent with the results of the other models. None of the extent variables in the project title was statistically significant, indicating that the extent of the features in the project title causes no discernible effect on fundraising success rate.

Moreover, we examined the impact of the extent variables in the project details in Model 5. As shown in Table 5, the control variables also significantly affected the fundraising results. In addition, the length of the description in the project details exhibited a significant negative effect on fundraising success. *DNMWords* and *DNNWords* emerged as positive predictors of project success, which demonstrates that the more money-related and negative emotion words there are in the project details, the higher the success rate. The coefficient of *DAge* (–2.11, *P*<.001) suggests that younger patients are more likely to fundraise successfully in medical crowdfunding. The coefficient of *DOccupation* was also negative, showing that for patients whose occupations are national civil servant, professional skill worker, clerk, business and service worker, solider, child, student, and public-spirited person, the success rate of fundraising will decrease sequentially.

**Table 5:** Regression results of extent variables.

Variables		Model 4 (adjusted R^2^=0.625)				Model 5 (adjusted R^2^=0.613)		
		*β*	*P* value	VIF^a^	*β*		*P* value	VIF
**Control**								
	Funding goal	–19.51	<.001	1.352	–17.76		<.001	1.796
	Donors	19.88	<.001	1.185	18.46		<.001	1.297
**Title^b^**								
	TLength	–12.29	.25	1.049	–4.73		.68	1.106
	TAge	–1.22	.31	1.242	1.01		.48	1.553
	TGender	–1.72	.29	1.056	–3.37		.23	1.243
	TDisease	8.41	.53	1.009	14.09		.54	2.056
	TLocation	–7.48	.07	1.005	–8.01		.05	1.010
	TOccupation	.33	.36	1.051	.62		.11	1.080
	TNNWords	–9.00	.47	1.039	–8.57		.49	1.064
	TNPWords	1.49	.82	1.025	2.68		.69	1.030
**Details**								
	DLength	—^c^	—	—	–11.73		.01	3.015
	DAge	—	—	—	–2.11		<.001	1.010
	DGender	—	—	—	.84		.67	1.192
	DDisease	—	—	—	–.43		.98	2.097
	DLocation	—	—	—	–.26		.74	1.115
	DOccupation	—	—	—	–3.36		.004	1.254
	DNMWords	—	—	—	5.78		.02	1.152
	DNNWords	—	—	—	5.21		.02	1.585
	DNPWords	—	—	—	–6.52		.08	2.568

^a^VIF: variance inflation factor.

^b^TNMWords was missing the correlation coefficient, and was therefore removed.

^c^—: not included in model.

### Robustness Test

We used two methods to test the robustness of these empirical results: increasing sample data from other platforms and eliminating specific samples.

First, we increased the sample data to verify whether the results will be affected by platform differences. We collected 382 crowdfunding projects from the “Fun in Funding” platform [55], which is also a well-known medical crowdfunding platform in China. We use the new sample dataset to reexamine Model 3, and the results are presented in Table 6; the regression results using the original sample data (Table 4) are also shown for comparison. In the project title, except for the presence of the patient’s disease that became insignificant, the conclusions of the remaining variables were consistent. In the project details, the presence of the location changed from insignificant in the original model to significant with the added data, and there was no difference in the results for the other variables.

Second, specific samples were eliminated to test whether they had a major driving effect on the results. There was a relatively large number of samples with a small success rate in the original dataset. Therefore, we removed the sample data below the average success rate (0.31) and used the remaining sample data to perform multiple regression analysis on Model 3. Table 6 shows that in the project title, the presence of the patient’s age and gender exhibited significant effects, coinciding with the previous conclusions. The presence of disease and location was significant at a 90% CI, which can also be statistically explained. The presence of occupation and negative emotions changed from significant to insignificant, whereas the presence of positive emotions transformed from insignificant to significant. By contrast, in the project details, the presence of money-related words became insignificant, and there was no change in the results for the other variables.

In summary, after using the two methods to complete the robustness test, we found that the results were mainly consistent with those obtained previously, indicating that our findings are relatively robust.

**Table 6:** Regression results of robustness tests.^a^

Variables			Model 1 (adjusted R^2^=0.643)							Model 2 (adjusted R^2^=0.590)							Model 3 (adjusted R^2^=0.254)				
			*β*		*P* value		VIF^b^		*β*			*P* value		VIF		*β*			*P* value		VIF
**Control**																					
	Funding goal	–20.53		<.001		1.179		–19.74			<.001		1.167		–17.25			<.001		2.599	
	Donors	18.90		<.001		1.097		17.62			<.001		1.097		11.41			<.001		2.555	
**Title**																					
	TPAge	–2.96		<.001		1.051		–2.11			.02		1.051		–3.76			.02		1.053	
	TPGender	–2.29		.001		1.043		–2.04			.003		1.038		–2.54			.04		1.057	
	TPDisease	1.41		.03		1.037		.90			.18		1.034		2.16			.07		1.054	
	TPLocation	–5.45		.05		1.009		–5.22			.07		1.008		–10.46			.03		1.021	
	TPOccupation	2.51		.02		1.025		2.34			.03		1.023		1.68			.33		1.031	
	TPMoney	–1.85		.68		1.011		–1.01			.83		1.011		2.93			.69		1.026	
	TPNWords	–3.76		.04		1.008		–4.84			.005		1.011		–1.24			.72		1.024	
	TPPWords	–1.02		.21		1.017		–.78			.34		1.018		–4.06			.007		1.038	
**Details**																					
	DPAge	–4.36		<.001		1.039		–4.89			<.001		1.033		–4.55			.003		1.081	
	DPLocation	–1.20		.17		1.031		–2.06			.02		1.029		0.36			.80		1.041	
	DPOccupation	3.07		.01		1.041		3.18			.01		1.034		4.48			.03		1.082	
	DPMoney	5.01		.02		1.004		6.11			.005		1.003		2.43			.65		1.013	
	DPNWords	2.55		.04		1.042		2.65			.03		1.037		6.51			.001		1.058	
	DPPWords	–34.65		<.001		1.013		–36.57			<.001		1.012		–14.92			.05		1.025	

^a^*P*<.10 at 90% CI is considered to be significant.

^b^VIF: variance inflation factor.

## Discussion

### Principal Findings

According to 4903 medical crowdfunding projects obtained from the Tencent Charity platform in China and using the theory of persuasion as the theoretical basis, this study applied multiple regression analysis to examine the impact of the existence variables and extent variables in the project title and project details on the success rate of medical crowdfunding. Owing to the requirements of the platform, all projects met the three aspects (authenticity, diagnosis, and charities) of credibility appeals; hence, we discuss the research results from two categories of appeals: rational and emotional appeals.

First, in rational appeals of the project title, the regression results of the existence variables showed that the presence of the patient’s disease and occupation had a positive impact on the success rate of the medical crowdfunding project. Providing a narrative of the patient’s disease and occupation allows potential donors to quickly understand who is raising money for what disease in a specific crowdfunding project. The presence of the patient’s age and gender had a negative impact on the success rate, indicating that the seeker may not need to describe these attributes of the fundraiser. In emotional appeals of the project title, the presence of negative words had a negative impact on the success rate, which shows that people do not wish to see the requesters’ negative emotions in the title description.

With respect to rational appeals of the project details, analysis of the existence variables suggested that the presence of a patient’s occupation and money-related words is positively correlated with the fundraising success rate. The patient’s occupation can arouse readers’ pity, and money-related words can accurately describe the economic plight of the requesters. Majumdar and Bose [20] also suggested that the presence of monetary evidence can promote the success of crowdfunding. The presence of the patient’s age was negatively correlated with the success rate, which indicates that the patient’s age does not need to be described in the project details. In emotional appeals of the project details, the presence of negative emotions exhibited a positive impact on the fundraising success rate. However, Majumdar and Bose [20] suggested that the presence of negative emotions had no significant effect because almost all requests describe the suffering of the seeker. We consider this difference to be related to differences in the primary concerns of people in different countries. Chinese donors intend to understand the distress that patients are suffering, which can only be expressed accurately with negative phrases. We found that the presence of positive emotions exhibited a negative effect on success rate, whereas Durand et al [23] proposed that positive emotions contributed to the success of organ transplantation crowdfunding projects. The reason for this difference may be that the physically disabled but optimistic image in crowdfunding for organ transplantation is more persuasive, which is not the case with crowdfunding for medical diseases.

Furthermore, we estimated the extent variables of the project’s textual features. The results reflect that in the project details, the patient’s age, occupation, and text length had a negative impact on the fundraising success rate, whereas the numbers of money-related words and negative emotions words had a positive impact. We further analyzed the causes of these results in detail. First, we suggest that the number of money-related words has a significant positive impact on the success rate of fundraising, which coincides with the findings of Majumdar and Bose [20] and Xu and Wang [24]. Second, we found that the number of negative phrases has a positive impact on the success rate, whereas Majumdar and Bose [20] came to the opposite conclusion. They believed that the excessive use of negative appeals may seem manipulative to the potential donor. The reason for this difference may be related to the different cultures of multiple countries, as Chinese donors may be more likely to be moved by descriptions of patients’ suffering that use more negative phrases. Xu and Wang [24] suggested that using a tragic narrative strategy can arouse the sympathy of Chinese potential donors. Third, we found that the length of the detailed narrative negatively affects the success rate, which is also inconsistent with the results of Majumdar and Bose [20]. The reason for this conflict may be that a long description can make people impatient and cause them to lose interest in donating. Iyengar and McGuire [56] suggested that humans are “cognitive misers,” meaning that they will predigest limited information and make a rapid decision according to the principle of least possible effort. Fourth, Liang et al [46] proposed that age has no significant impact on reward-based crowdfunding, whereas our results show that young patients are more likely to succeed in medical crowdfunding because they more easily arouse people’s sympathy. Similar to this conclusion, Ren et al [57] found that people are generally more generous with fundraising projects targeting children than adults. Finally, our study suggests that in the project details narrative of medical crowdfunding, for patients whose occupations are national civil servant, professional skill worker, clerk, business and service worker, solider, child, student, and public-spirited person, the success rate of fundraising will decrease sequentially.

Our results have clear implications with respect to the impact of the funding goal and donors as control variables. These two variables had evident significant effects on the fundraising success rate. Specifically, the funding goal was negatively correlated with the success rate, which is in accordance with the conclusions of Gorbatai and Nelson [51] and Kim et al [48], whereas the number of donors had a positive impact on the success rate, coinciding with the results of Aleksina et al [44], which revealed that sharing or forwarding information about crowdfunding activities on social networks can result in more financial support and an increased fundraising success rate.

### Theoretical Implications

This study makes the following four theoretical contributions. First, this paper enriches and expands research in the realm of medical crowdfunding. The success rate of medical crowdfunding worldwide is generally low at present, yet few previous studies have directly explored the impact of textual features on the success rate of medical crowdfunding projects. This study used real projects to study the roles of textual features of the project title and project details, and the findings can provide a reference for researchers in medical crowdfunding.

Second, this study supplements and improves the online crowdfunding literature. Existing studies have mainly discussed the effect of the project details features but ignored the project title. By examining the project title features, we found that the presence of a patient’s disease and occupation has a significant positive impact on the success rate of fundraising, whereas the presence of age, gender, and negative words has a negative impact. Thus, the role of the project title cannot be overlooked.

Third, the results of this study also contribute to the literature on persuasion theory. Although there have been many studies on human behavior based on the theory of persuasion, this study is the first to use this theory to explore the effects of textual persuasion strategies in the field of medical crowdfunding. Specifically, there is very little research on the persuasive strategy of the project title in online crowdfunding, although we discovered that this also has a contribution to fundraising results. For rational appeals, the presence of the patient’s age, gender, disease, and occupation in the project title exhibited significant effects on the success rate of fundraising, whereas the presence of monetary evidence exhibited no important effect. For emotional appeals, the presence of negative emotions in the project title played a negative role on the success rate, whereas the presence of positive emotions had an insignificant effect. Moreover, the persuasion strategy of the project details in medical crowdfunding also differs from that in other types of crowdfunding. Liang et al [46] proposed that age has no significant impact on reward-based crowdfunding, whereas our results showed that young patients are more likely to successfully raise funds. There is no literature proposing that occupation has an important impact, although our results indicated that the presence of the patient’s occupation makes the project more persuasive. Consistent with the findings of Majumdar and Bose [20], we suggest that the presence of monetary evidence can better persuade people to donate, and this persuasion effect increases with a greater frequency of that presence. For emotional appeals, the persuasion strategy of medical crowdfunding also diverges from that of other crowdfunding types. Majumdar and Bose [20] indicated that the presence of negative emotions had no significant effect on obtaining pizza donations. Durand et al [23] discovered that positive emotions contribute to the success of organ transplantation crowdfunding projects. However, our results indicate that the presence of negative emotions and positive emotions significantly affect the success rate of crowdfunding, but that the former affects the rate positively while the latter affects it negatively.

Fourth, this paper is beneficial for the online donation literature. In recent years, many studies have explained the donation behaviors of online users through external factors such as the celebrity effect and individual factors [30,58,59]. Behl and Dutta [60] also found that gamification has a positive impact on donor behavior on crowdfunding platforms for disaster relief operations. This study delineates how textual features in medical crowdfunding affect users’ online donation behaviors, showing that the presence of a patient’s disease and occupation in the project title has a positive relationship with the fundraising success rate, whereas the presence of age, gender, and negative words has a negative relationship. Moreover, the presence of the patient’s occupation, monetary evidence, and negative emotions in the project details plays a positive role in the success rate, whereas the presence of the patient’s age and positive emotions plays a negative role. Therefore, these features of the crowdfunding text may also affect the donation behaviors of online users. The conclusions can therefore provide assistance for research in the field of online donations.

### Practical Implications

On the basis of our findings and previous research, we propose three implications for fundraisers from two categories (rational and emotional appeals) of the theory of persuasion and the three perspectives of the project title, project details, and nontextual features. First, for rational appeals of the project title, the results show that the presence of the patient’s disease and occupation is positively related to the success rate of the medical crowdfunding project; thus, fundraisers should be advised to clearly describe the patient’s illness and occupation in the project title. We also found that the presence of patient’s age and gender is negatively related to the success rate, implying that age and gender are unnecessary in the narrative of the project title. For emotional appeals of the project title, the presence of negative words has a negative impact on the success rate, which suggests that the requester does not need to include negative emotions in the description of the project title.

Second, for rational appeals of the project details, the results indicate that the presence of a patient’s occupation and money-related words has a positive impact on the fundraising success rate, which suggests that the description can include information about the patient’s occupation and finances. Projects that use more money-related words are more likely to succeed, and the success rate will gradually decrease when the patient’s occupation is national civil servant, professional skill worker, clerk, business and service worker, solider, child, student, and public-spirited person. In addition, the presence of the patient’s age has a negative impact on the success rate, implying that the patient’s age can be omitted from the description of the project details. However, if the patient is young, this information should be stated in the project details. For emotional appeals of the project details, the presence of negative words has a positive impact on the fundraising success rate, while the presence of positive words has the opposite effect, indicating that using as many negative phrases as possible is conducive to promoting fundraising success. The last suggestion is that the description in the details should be concise and powerful, and should not be too long.

Third, our investigation of the control variables showed that the fundraising success rate increases with increases in the number of donors but decreases with increases in the funding goal. This finding implies that requesters need to set appropriate goals when raising funds, and should widely disseminate and share their projects on social networks to seek more donations instead of forwarding the projects only to relatives and friends.

Finally, our study also has important implications for crowdfunding platform managers. Specifically, they can use a method similar to that presented herein to explore factors that are of concern to users in other types of crowdfunding text narratives. The managers of medical crowdfunding platforms may take our conclusions as a reference and provide proposals for fundraisers who have no idea how to create the crowdfunding text. Additionally, we recommend that some tags be intercalated in crowdfunding projects to highlight critical information that will be beneficial to the user experience of both fundraisers and donors.

### Limitations and Future Research

Although this study makes several contributions, there are still some limitations that need to be recognized. First, our work considered the effects of textual features on the success of medical crowdfunding and did not examine noncontent features other than the funding goal and donors, such as the number of updates. Second, the process of feature extraction was somewhat cumbersome and time-consuming, and should be improved in the future. For example, future research can consider using machine learning and artificial intelligence to improve the efficiency of feature extraction. Third, this study collected only data related to the crowdfunding projects and excluded donor information. Future research can include donor information data and further analyze the impact of textual features on different donors. Fourth, this study only used fundraising projects from a Chinese crowdfunding platform, and therefore it is unclear whether the conclusions will be applicable to crowdfunding platforms from other countries. Thus, future research should be extended to collect data from multiple crowdfunding platforms in different countries for exploring the differences between crowdfunding in China and other countries from a cultural perspective. Finally, this study did not compare the effect of credibility appeals because of the limitation of the dataset.

### Conclusion

This study focused on the effect of the textual features of medical crowdfunding projects on their success rate, taking 4903 real projects collected from the Tencent Charity platform in China as the research sample. We developed our research model according to previous studies and Aristotle’s theory of persuasion. We further divided the textual features of the project title and project details into three categories of rational, emotional, and credibility appeals, which were analyzed from two perspectives of existence and extent. We show how some of the persuasive appeals and nontextual cues have a significant impact on fundraising success rates. The results show that in addition to the project details, the features of the project title also have an important impact on the success rate of fundraising. These findings provide important theoretical and managerial implications for medical crowdfunding. Additionally, our research will be beneficial to all donation-based charity platforms and requesters with medical fundraising needs.

## Appendix

Multimedia Appendix 1 The pseudocode of the crawler program.

Multimedia Appendix 2 Quantification of location variables.

Multimedia Appendix 3 Quantification of occupation variables.

Multimedia Appendix 4 Correlation of existence variables.

Multimedia Appendix 5 Correlation of extent variables.
