# Supplementary material for: Effect of Textual Features on the Success of Medical Crowdfunding: Model Development and Econometric Analysis from the Tencent Charity Platform
Source: J Med Internet Res. 2021 Jun 11;23(6):e22395. doi: 10.2196/22395 (PMC8235274; doi:10.2196/22395)
Supplement: Multimedia Appendix 1 [file jmir_v23i6e22395_app1.pdf]

Multimedia Appendix 1. The pseudocode of the crawler program

Input: *Iinitial_url*: URL of the first page of the completed medical crowdfunding project

N: The number of sample data

Output: *TC_Data*: The medical crowdfunding projects from Tencent Charity

while *count* < *N* **do**

    Add the next page's url of *Initial_url* to *Initial_url_list*

    count += 1

end while

for each $initial\_url_i$ of *Initial_url_list* **do**

    Get *detail_page_list* of the $initial\_url_i$

    for each $detail\_page_j$ of *detail_page_list* **do**

        Add the *Project_title*, *Project_details*, *Funding goal*, *Donors*, *Fundraising amount* and *Charity* of $detail\_page_j$ to *TC_Data*

    end

end
