# Supplementary material for: Effect of Textual Features on the Success of Medical Crowdfunding: Model Development and Econometric Analysis from the Tencent Charity Platform
Source: J Med Internet Res. 2021 Jun 11;23(6):e22395. doi: 10.2196/22395 (PMC8235274; doi:10.2196/22395)
Supplement: Multimedia Appendix 2 [file jmir_v23i6e22395_app2.pdf]

Multimedia Appendix 2. Quantification of location variables

Location	Per capita GDP in 2018 ($)	Quantification
Beijing	21188	1
Shanghai	20421	2
Tianjin	18021	3
Jiangsu	17445	4
Zhejiang	14907	5
Fujian	13838	6
Guangdong	13058	7
Shandong	11525	8
Inner Mongolia	10322	9
Hubei	10079	10
Chongqing	10007	11
Shaanxi	9769	12
Liaoning	9593	13
Jilin	8748	14
Ningxia	8175	15
Hunan	8001	16
Hainan	7851	17
Henan	7577	18
Xinjiang	7567	19
Sichuan	7387	20
Anhui	7250	21
Hebei	7219	22
Qinghai	7207	23
Jiangxi	7193	24
Shanxi	6850	25
Heilongjiang	6529	26
Tibet	6500	27
Guangxi	6270	28
Guizhou	6233	29
Yunnan	5629	30
Gansu	4735	31
