# Supplementary material for: Effect of Textual Features on the Success of Medical Crowdfunding: Model Development and Econometric Analysis from the Tencent Charity Platform
Source: J Med Internet Res. 2021 Jun 11;23(6):e22395. doi: 10.2196/22395 (PMC8235274; doi:10.2196/22395)
Supplement: Multimedia Appendix 3 [file jmir_v23i6e22395_app3.pdf]

Multimedia Appendix 3. Quantification of occupation variables

Occupation	Quantification
National civil	1
Professional skill worker	2
Clerk	3
Business and service worker	4
Solider	5
Child	6
Student	7
Public-spirited person	8
Others	9
