# Supplementary material for: Effect of Textual Features on the Success of Medical Crowdfunding: Model Development and Econometric Analysis from the Tencent Charity Platform
Source: J Med Internet Res. 2021 Jun 11;23(6):e22395. doi: 10.2196/22395 (PMC8235274; doi:10.2196/22395)
Supplement: Multimedia Appendix 4 [file jmir_v23i6e22395_app4.pdf]

Multimedia Appendix 4. Correlation of existence variables

Variables	[1]	[2]	[3]	[4]	[5]	[6]	[7]	[8]	[9]	[10]	[11]	[12]	[13]	[14]	[15]	[16]
[1]Funding goal	1															
[2]Donors	.27	1														
[3]TPAge	-.09	.02	1													
[4]TPGender	.12	.04	.01	1												
[5]TPDisease	-.08	-.01	.09	.08	1											
[6]TPLocation	-.02	.01	-.03	.04	-.02	1										
[7]TPOccupation	-.00	.06	.11	.01	.07	.02	1									
[8]TPMoney	-.05	-.01	-.03	-.03	-.03	-.01	-.01	1								
[9]TPNWords	.02	.01	.00	.02	.05	-.02	-.00	-.01	1							
[10]TPPWords	.03	-.01	-.07	-.04	-.06	-.01	-.02	-.03	.07	1						
[11]DPAge	-.06	-.07	.10	-.05	.05	-.02	-.01	.00	.01	.01	1					
[12]DPLocation	.14	-.02	-.02	.05	-.04	.03	.02	-.00	.00	.02	.04	1				
[13]DPOccupation	.02	-.05	.09	-.09	.03	-.03	.05	.02	.00	-.01	.13	.02	1			
[14]DPMoney	-.00	.03	-.00	-.00	-.00	.02	-.01	-.01	.01	.02	.01	.02	.03	1		
[15]DPNWords	.19	.07	-.01	.07	-.04	.01	-.01	-.03	.00	-.00	-.00	.04	.01	.00	1	
[16]DPPWords	.08	.00	.02	.01	-.01	-.04	-.00	-.07	.01	-.01	.01	.01	.01	-.01	.02	1
