# Supplementary material for: Effect of Textual Features on the Success of Medical Crowdfunding: Model Development and Econometric Analysis from the Tencent Charity Platform
Source: J Med Internet Res. 2021 Jun 11;23(6):e22395. doi: 10.2196/22395 (PMC8235274; doi:10.2196/22395)
Supplement: Multimedia Appendix 5 [file jmir_v23i6e22395_app5.pdf]

Multimedia Appendix 5. Correlation of extent variables

Variables	[1]	[2]	[3]	[4]	[5]	[6]	[7]	[8]	[9]	[10]	[11]	[12]	[13]	[14]	[15]	[16]	[17]	[18]
[1]TLength	1																	
[2]TAge	.17	1																
[3]TGender	.05	.13	1															
[4]TDisease	-.00	-.04	-.01	1														
[5]TLocation	.01	.02	.01	-.01	1													
[6]TOccupation	.06	-.15	.01	.00	.03	1												
[7]TNMWords	.04	-.02	-.01	-.01	-.01	-.01	1											
[8]TNNWords	.02	-.01	.01	-.00	-.02	-.00	-.01	1										
[9]TNPWords	-.01	.01	-.02	-.00	-.01	-.02	-.03	.07	1									
[10]DLength	.21	.19	.04	-.07	-.01	-.01	-.06	.03	.02	1								
[11]DAge	.05	.07	.12	.02	.00	-.04	-.03	.00	.05	.23	1							
[12]DGender	-.01	-.08	.34	.01	-.01	.06	.00	.00	.00	.00	-.01	1						
[13]DDisease	-.03	.06	-.02	.52	.01	.00	.01	-.00	.02	-.03	.07	.01	1					
[14]DLocation	.04	.09	.00	-.02	.06	.01	.01	.00	.00	.12	.08	-.03	-.01	1				
[15]DOccupation	.00	.42	.04	-.00	.01	.01	-.01	.03	-.01	.03	.28	-.04	.02	.03	1			
[16]DNMWords	.05	-.04	.00	.00	.00	-.00	-.01	.01	.01	.20	.01	-.02	.04	.03	-.05	1		
[17]DNNWords	.09	.12	.04	-.04	-.01	-.02	-.02	.03	.02	.53	.11	-.03	-.04	.06	.02	.07	1	
[18]DNPWords	.17	.17	.04	-.06	-.02	-.01	-.04	.02	.04	.79	.23	-.00	-.07	.07	.04	.14	.49	1
